# Load-induced deformation of the tibia and its effect on implant loosening detection

**DOI:** 10.1038/s41598-023-49177-z

**Published:** 2023-12-08

**Authors:** M. A. ter Wee, J. G. G. Dobbe, G. S. Buijs, A. J. Kievit, M. U. Schafroth, M. Maas, L. Blankevoort, G. J. Streekstra

**Affiliations:** 1 Department of Biomedical Engineering and Physics, Amsterdam UMC Location University of Amsterdam, Meibergdreef 9, Amsterdam, The Netherlands; 2 Amsterdam Movement Sciences, Musculoskeletal Health, Amsterdam, The Netherlands; 3 Department of Orthopedic Surgery and Sports Medicine, Amsterdam UMC Location University of Amsterdam, Meibergdreef 9, Amsterdam, The Netherlands; 4 Department of Radiology, Amsterdam UMC Location University of Amsterdam, Meibergdreef 9, Amsterdam, The Netherlands

**Keywords:** Computed tomography, Biomedical engineering, Bone

## Abstract

CT imaging under external valgus and varus loading conditions and consecutive image analysis can be used to detect tibial implant loosening after total knee arthroplasty. However, the applied load causes the tibia to deform, which could result in an overestimation of implant displacement. This research evaluates the extent of tibia deformation and its effect on measuring implant displacement. Ten cadaver specimen with TKA were CT-scanned under valgus/varus loading (20 Nm), first implanted without bone cement fixation (mimicking a loose implant) and subsequently with bone cement fixation (mimicking a fixed implant). By means of image analysis, three relative displacements were assessed: (1) between the proximal and distal tibia (measure of deformation), (2) between the implant and the whole tibia (including potential deformation effect) and (3) between the implant and the proximal tibia (reduced deformation effect). Relative displacements were quantified in terms of translations along, and rotations about the axes of a local coordinate system. As a measure of deformation, the proximal tibia moved relative to the distal tibia by, on average 1.27 mm (± 0.50 mm) and 0.64° (± 0.25°). Deformation caused an overestimation of implant displacement in the cemented implant. The implant displaced with respect to the whole tibia by 0.45 mm (± 0.22 mm) and 0.79° (± 0.38°). Relative to the proximal tibia, the implant moved by 0.23 mm (± 0.10 mm) and 0.62° (± 0.34°). The differentiation between loose and fixed implants improved when tibia deformation was compensated for by using the proximal tibia rather than the whole tibia.

## Introduction

Following total knee arthroplasty (TKA), approximately 20% of the patients are not satisfied with their outcomes because of pain, stiffness, instability and other reasons^1^. Diagnosing the cause of these complaints is an important consideration to determine if revision surgery is required, or conservative treatment (physiotherapy, patient education, pain medication etc.) suffices. In the majority of the patients that undergo revision surgery, it concerns loosening of the tibial component, yet a reliable diagnostic tool is currently unavailable^2^. Current diagnostic imaging methods to detect implant loosening that are based on the assessment of geometric features in static images, such as radiography and nuclear imaging, are often inconclusive. Radiography is prone to variability in execution of the imaging protocol and image evaluation^3,4^ and shows a moderate diagnostic accuracy^5^. Positron emission tomography and single photon emission computed tomography combined with computed tomography (CT) are expensive methods and their diagnostic accuracies are ambiguous (between 56 and 100% sensitivity and specificity)^6^. Load-induced or long-term follow-up roentgen stereophotogrammatric analysis (RSA) can detect submillimeter implant migration and displacement, but requires the invasive insertion of bone markers and is therefore not suitable for general clinical practice^7,8^.

**Figure 1:**
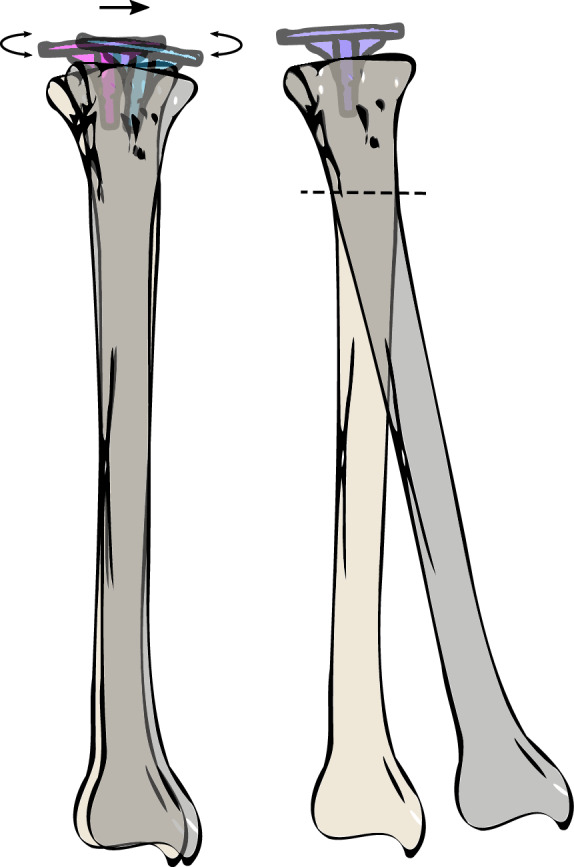
Graphical representation of the effect of tibia deformation (exaggerated for clarity) on implant displacement assessment of a fixed implant, measured relative to the whole tibia (left) or relative to the proximal tibia (right). As a result of deformation, the tibia model segmented in valgus does not align with the tibia in varus position and registers to an intermediate position. The apparent implant displacement is caused by a registration mismatch due to tibia deformation. If the proximal tibia is used as reference object, the registration mismatch may be reduced.

A method was recently reported to measure implant displacement using CT image analysis^9,10^, similar to measuring load-induced implant micro-motion using RSA or CT^11–17^. This non-invasive technique uses CT images of the knee under valgus and varus load. The loads induce a displacement between the tibial implant and the tibia bone. The implant displacement is subsequently quantified using image analysis techniques. The implant and tibia are segmented in the valgus scan and rigidly registered to the varus scan. However, under the applied load, the bone itself may deform, which provokes slightly different shapes of the tibia in valgus and varus position. This can be expected to affect registration of the tibia model and therefore the observed displacement between the implant and bone may appear larger. An exaggerated illustration of the deformation mismatch of the tibia model with a fixed implant is shown in Fig. 1. If the implant displacement is measured relative to the whole tibia (Fig. 1 left), the alignment of the tibia model in valgus, by registration, deviates from the varus model (target), which contributes to erroneous observation of implant displacement. However, the effect of deformation may be reduced by evaluating implant displacement with respect to a smaller proximal part of the tibia (Fig. 1 right).

It is hypothesized that exerting an external valgus-varus loading to the knee causes the tibia to deform, leading to an overestimation of implant displacement measurement. The research questions are: 1. To what extent does tibia deformation affect implant displacement measurement? 2. How much do cadaver tibiae deform under valgus-varus loading? 3. Does the distinction between fixed- and loose implants improve when the effect of deformation is reduced by quantifying implant displacement with respect to the proximal tibia instead of the whole tibia?

## Methods

**Figure 2:**
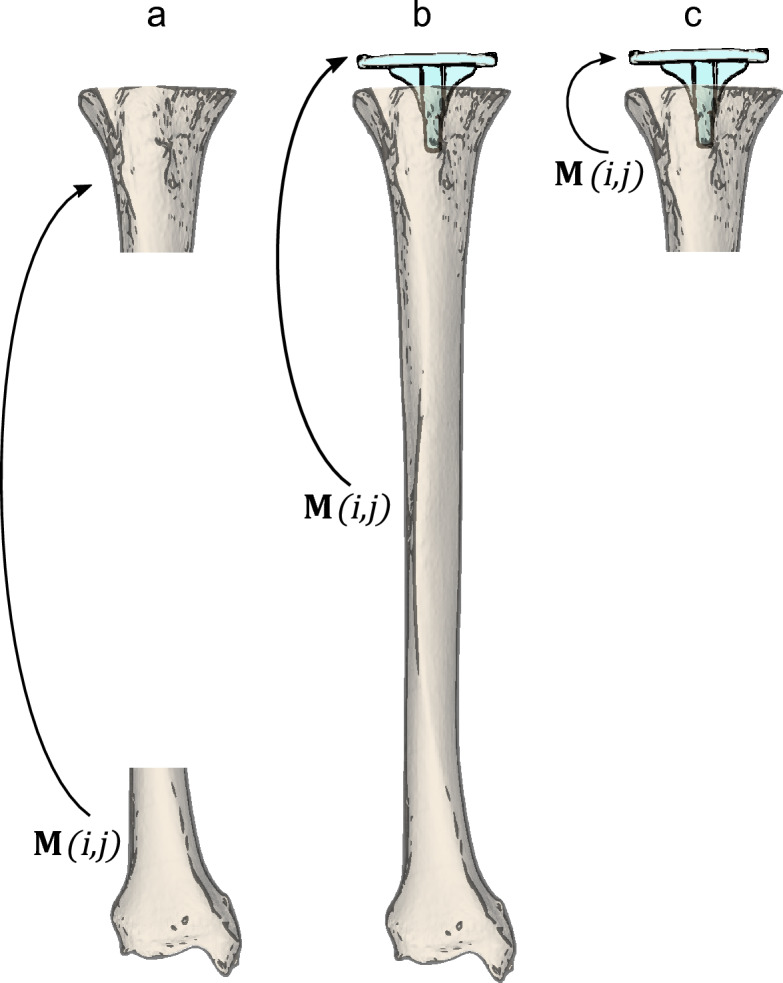
Overview of the investigated relative displacements. (**a**) The proximal tibia with respect to the distal tibia to evaluate deformation. (**b**) Implant displacement with respect to the whole (100%) tibia below the implant plateau, and (**c**) with respect to the (20%) proximal tibia, to evaluate the effect of tibia deformation on implant displacement measurement. The matrix $\mathbf{M}\left(i,j\right)$ represents the relative displacement between the objects.

In this study, CT scans of cadaver specimens from an earlier study^10^ were used to evaluate tibia deformation by quantifying the relative displacement between the proximal and distal tibia (Fig. 2a). Implant displacement is quantified with respect to the whole tibia segment below the implant plateau (more affected by deformation, Fig. 2b) and with respect to 20% of that tibia segment (less affected by deformation, Fig. 2c) to evaluate whether tibia deformation affects the measurement of implant displacement, and to investigate whether reducing the effect of deformation is beneficial for determining a threshold to establish implant loosening.

In the remainder of this paper, when evaluating the effect of deformation, the reference is to either using the "whole tibia" (Fig. 2b) or “proximal tibia” (Fig. 2c).

### Data

All CT scans in this study included part of the femur, the knee and the lower leg, and were acquired with a Brilliance 64-channel CT scanner (Philips Healthcare, Best, The Netherlands; 160 mAs, 120 kV, convolution kernel D, isotropic voxel spacing of 0.45 mm). The study data concerned three left sided and seven right sided legs with ages ranging between 68 and 92 years (median 82 years), six males and four females. The cadaver specimens were obtained through the body donation program from the Department of Medical Biology of the Amsterdam UMC, location Academic Medical Center, in The Netherlands. The bodies from which the samples were taken were donated to science in accordance with Dutch legislation and the regulations of the medical ethical committee of the Amsterdam UMC, location Academic Medical Center. This procedure of body donation and subsequent use of these bodies for scientific research is in accordance with Dutch funeral legislation (WLB: Article 1 and Article 67).

For assessment of the methodological error, one frozen cadaver specimen with a TKA implant was scanned repeatedly (n = 10) in different positions and orientations within the scanner, without external loading to exclude a potential effect of implant motion or tibia deformation.

**Figure 3:**
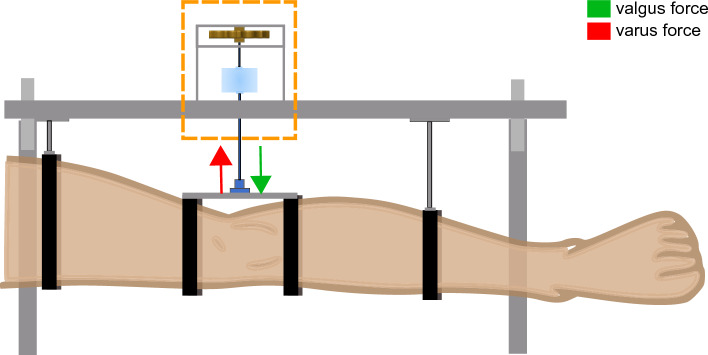
Schematic representation of the loading device^9^ used to apply a moment to the knee to bring it to valgus position (actuator, orange box, pushes; green arrows) or varus position (actuator pulls; red arrows) to exert displacement forces to the implant during CT acquisition.

To evaluate the degree of tibia deformation and its effect on measuring implant displacement, 10 cadaver specimens were fitted with an implant (Vanguard, Zimmer Biomet, Warsaw, Indiana, United states^18^). First, the implant was placed without cement by press fitting and manually moving the tibial component around to mimic bone resorption hereby imitating a loose implant. The leg was then CT scanned under a valgus- and varus load of 20 Nm using a loading device^9^ (Fig. 3), which loads the leg in a four-point bending configuration with a uniform moment over the knee, independent of its size. Subsequently, the implants were removed and replaced using bone cement to imitate fixed implants and the imaging protocol was repeated. This resulted in 40 CT scans, 10 × 2 uncemented implants in cadaver legs with valgus and varus load, 10 × 2 cemented implants in cadaver legs with valgus and varus load.

### Image analysis

Custom software for 3D image analysis was used to quantify relative bone and implant displacements between the two CT scans^19^. The implant and the tibia cortex were segmented from the valgus image using a Laplacian level-set segmentation growth algorithm^20^, which was initialized by threshold-connected region growing including voxels above an empirically chosen intensity threshold (approx. 1600 for tibia cortex, 3500 for the implant). A polygon mesh of the surface of the object was extracted using the marching cubes algorithm for visualization and registration purposes^21^. The gray-level image was subsequently interpolated and sampled at + 0.3 mm and − 0.3 mm along the surface normal of each point of this polygonal mesh, resulting in a double-contour polygon with gray-level values from the valgus image assigned to the points. The Nelder-Mead downhill simplex optimization algorithm was subsequently used to find the position of the same object in the varus image^22^. To this end the correlation coefficient between gray-level values assigned to the double-contour mesh points, and the varus image, served as optimization metric^23,24^. Registration occurred in a six-parameter search space: translations along the x, y, z axes and rotations about these axes (φ_X_, φ_Y_, φ_Z_; sequence: y, x, z).

The absolute displacement of the first object (implant or proximal tibia; Fig. 2) between the valgus and varus scan is described by matrix *M*_*i*_*.* The second object displacement (whole tibia, proximal tibia, or distal tibia; Fig. 2) is described by *M*_*j*_. The matrix representing the relative displacement, *M(i,j)* in the global coordinate system (the CT coordinate system) can now be calculated:

1 $$M\left(i,j\right)= {M}_{j}^{-1}{M}_{i}.$$

Relative implant displacement was expressed in a local coordinate system (CS), of which the origin was located in the segmentation image (valgus) at the centroid of the implant plateau surface, as discussed below. Its definition enables identifying displacements independently of the position of the leg inside the scanner. Moreover, the displacements parameters, expressed in terms of this CS, correspond to the observations of the surgeon. The CS is placed automatically, ruling out potential observer variability associated with manual placement of the anatomical landmarks used in the ISB convention^25^.

**Figure 4:**
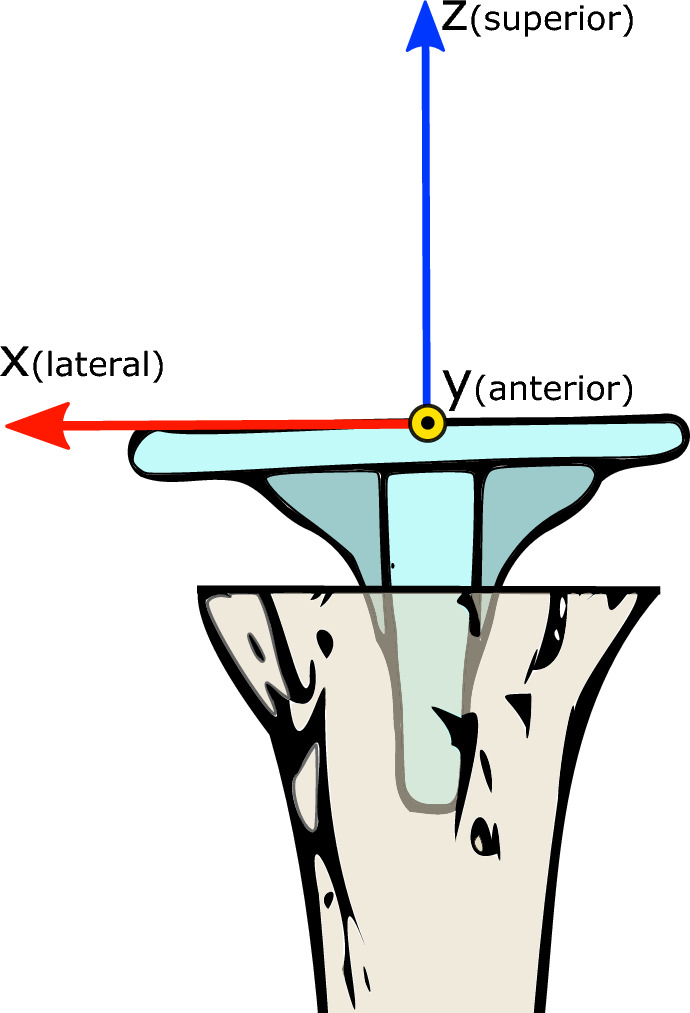
Ventral view of the proximal tibia with tibial implant. The local CS, in which the relative displacement between the objects was expressed, is defined as follows: The z-axis in the distoproximal direction, the x-axis points to the lateral side, and the y-axis is perpendicular to the x- and z-axes (posterior-anterior direction). The centroid of the implant plateau is used as center of rotation, and is placed at the origin of the fixed local CS, which was defined for the implant in the valgus image.

The location and orientation of the local CS was determined by projecting an axial grid of points at maximum z-position onto the implant plateau, and determining the principal axes of this projection using eigenvector analysis of the inertia tensor^26^. The axis pointing in the proximal direction represented the z-axis, the x-axis was chosen to the lateral side and the y-axis perpendicular to x and z (posterior-anterior direction, Fig. 4). To﻿ express the relative displacement between the mesh objects in terms of this local CS, the transformation matrix *M*_*CS*_ was defined which transformed the global CS to the local CS, so that the relative displacement of objects *i* and *j* (Eq. 1) is now given by^19^:

2 $${M}_{local}\left(i,j\right)={M}_{CS}^{-1}M\left(i,j\right){M}_{CS}.$$

Using the local coordinate system three translation (∆x, ∆y, ∆z) parameters were derived along the x, y, and z axes of the local CS, and three rotation parameters (∆φ_X_, ∆φ_Y_, ∆φ_Z_) about the local x, y and z axes.

The results of the (apparent) relative object displacement were expressed in three translation (x, y, z)- and three rotation (φ_X_, φ_Y_, φ_Z_) parameters, as well as the magnitude of translation and rotation, given by $\sqrt{{x}^{2}+{y}^{2}+{z}^{2}}$ and $\sqrt{{\mathrm{\varphi }}_{x}^{2}+{\mathrm{\varphi }}_{y}^{2}+{\mathrm{\varphi }}_{z}^{2}}$^27^.

Additionally, two scalar quantities, which are often used in clinical literature, were calculated to compare implant displacement of loose- and fixed implants: (1) a standard measure of implant migration in RSA studies, the maximum total point motion (MTPM), which is the motion in millimeters of a point on the implant that migrates the most^28^, (2) the mean target registration error (mTRE), which is defined as the mean distance between the initial (segmented) and the registered position of target features (i.e. landmark points)^29^. In our application it describes the average absolute translation of the points on the implant between valgus- and varus position. Both MTPM and mTRE combine the effect of object geometry and motion in one scalar quantity describing displacement.

### Experiments

Three experiments were conducted in this study to evaluate: (1) the methodological error of displacement measurement, (2) tibia deformation under valgus-varus loading and (3) implant displacement under valgus-varus loading.

#### Methodological error

The methodological error was separately assessed for the three relative object displacements (Fig. 2a–c) since the shape and size of each segmented object was different. The methodological error was assessed on the repeatedly (n = 10) scanned frozen cadaver specimen as follows: In each of the 10 cadaver scans, the implant and tibia were segmented, and the tibia was clipped at 20% of the length to collect the proximal and distal tibia segments. These object positions served as the reference position. Subsequently, the objects (implant, distal segment, proximal segment and whole tibia) were registered from the reference position in the segmented image to the residual nine scans. This procedure was repeated by using a different scan each time for segmentation, and registration to the remaining nine scans. If any relative displacement is observed, it can be attributed to methodological error caused by scanning and analysis. The apparent relative displacement thus observed between the objects in all the registration images was calculated and compared with the relative position in the corresponding segmentation image. Therefore, for each relative displacement evaluation, 2 × 90 registrations were involved, resulting in 90 apparent relative displacements. The average of these values served to represent the accuracy of zero-displacement, the standard deviation was used as measure of precision.

#### Tibia deformation under load

Tibia deformation between valgus and varus load was quantified in the 10 cadaver specimens (with cemented implants) as follows: The tibia cortices were segmented in the valgus scans and the proximal and distal bone segments were clipped at 20% of the length. These segments were registered to the varus scan and their relative displacement expressed in terms of a local CS (see section Image analysis and Fig. 4). The relative displacement of the distal tibia with respect to the proximal tibia between valgus and varus was attributed to tibia deformation.

#### Implant displacement under load

The effect of tibia deformation on measuring implant displacement in the cadaver specimens was evaluated by calculating the implant displacement with respect to the whole tibia (deformation effect expected) and with respect to the proximal of the tibia (smaller deformation effect expected). The tibia implant and tibia were segmented in the valgus scan and registered to the varus scan. The relative implant displacements with respect to the proximal- and to the whole tibia were quantified. These evaluations were performed for the CT scans of the cadavers, with cemented and uncemented implants. The results of the cemented implants were compared with the tibia deformation results, since the implant displacement was assumed to be minimal and therefore the deformation effect was expected to be more apparent.

The results of the cemented and uncemented implants were used to assess the differentiation between fixed and loose implants when using the whole or proximal tibia as the reference object.

### Statistical analysis

Since the number of experiments was limited (10 cadavers), it was assumed that the data were not normally distributed.

A non-parametric rank test (Mann–Whitney *U* test) was performed to test if tibia deformation displacements and implant displacement measured with respect to the whole or proximal tibia were statistically significantly larger than the methodological error of the measurement.

A Wilcoxon signed-rank test was used to test whether the decomposed translation and rotation parameters were statistically significantly larger than the methodological error. The methodological error was given by a conservative estimation of the maximum absolute error.

A paired Wilcoxon signed-rank test was used to test whether there was a statistically significantly difference between the methodological error of implant displacement measurement with respect to the whole or proximal tibia. This same test was used to test whether implant displacement, measured with respect to the whole tibia, was statistically significantly larger, due to deformation, compared to using the proximal tibia.

The level of statistical significance was set at α < 0.05. All statistical tests were conducted using JASP (Version 0.16.1)^30^.

## Results

### Methodological error

The accuracy and precision of the measurements of the three relative object replacements (Fig. 2a–c) are shown in Tables 1 and 2. The methodological errors are also plotted in the boxplots showing tibia deformation (Figs. 6 and 7) and implant displacement (Figs. 9 and 10) for easy comparison.

**Table 1:** Methodological error of tibia deformation measurement.

Tibia deformation	Accuracy	Precision
Translation magnitude [mm]	0.17	0.12
X translation [mm]	− 0.02	0.06
Y translation [mm]	0.12	0.14
Z translation [mm]	0.05	0.08
Rotation magnitude [°]	0.05	0.03
X rotation [°]	0.02	0.03
Y rotation [°]	0.00	0.02
Z rotation [°]	− 0.04	0.03

**Table 2:** Methodological error of implant displacement measurement relative to whole- and proximal tibia.

Implant displacement	Whole tibia		Proximal tibia		p-value
	Accuracy	Precision	Accuracy	Precision	
Translation magnitude [mm]	0.04	0.03	0.07	0.06	< 0.001
X translation [mm]	0.00	0.01	0.00	0.01	0.094
Y translation [mm]	0.01	0.01	− 0.02	0.03	< 0.001
Z translation [mm]	0.01	0.04	0.06	0.06	< 0.001
Rotation magnitude [°]	0.08	0.04	0.08	0.03	0.384
X rotation [°]	− 0.01	0.05	0.01	0.05	< 0.001
Y rotation [°]	0.00	0.04	0.00	0.05	0.933
Z rotation [°]	0.00	0.06	− 0.01	0.05	< 0.001
mTRE	0.05	0.02	0.08	0.06	< 0.001
MTPM	0.07	0.04	0.11	0.06	< 0.001

The p-values show the level of significance between the Whole and Prox tibia in each displacement parameter.

### Tibia deformation under load

**Figure 5:**
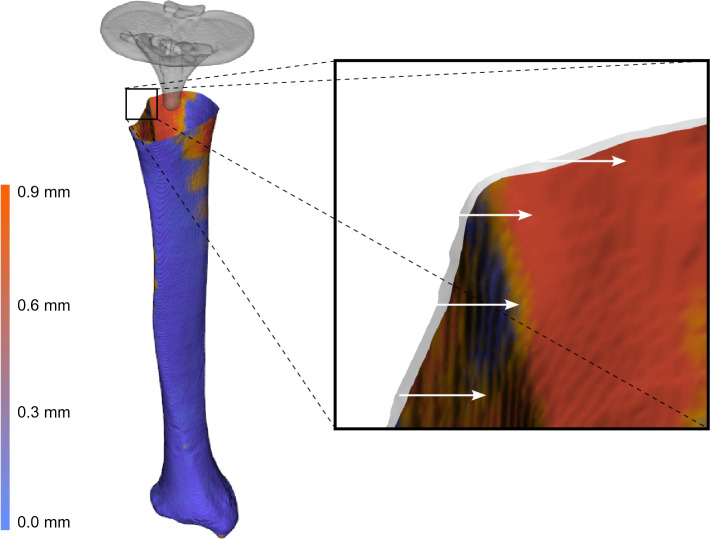
Visualization of the deformation between the tibia object segmented in the valgus scan (white) and the tibia segmented in the varus scan (heatmap), after aligning the distal 20% parts. The heatmap demonstrates the (nearest neighbor^20^) distance between the tibia segmentations. The white arrows indicate the direction of displacement between the valgus- and varus models, a result of tibia deformation.

A heatmap with colors that indicate the distance between the tibia in valgus and in varus position of one cadaver specimen was constructed by aligning two tibia segmentations, one in valgus position and one in varus position, using the transformation matrix obtained from registration of the distal 20% part. This illustrates that the deformation is typically found in the proximal tibia (Fig. 5). Zooming in on the proximal tibia, the mismatch between the proximal tibia in varus (light-grey contour) and valgus is clearly visible.

**Figure 6:**
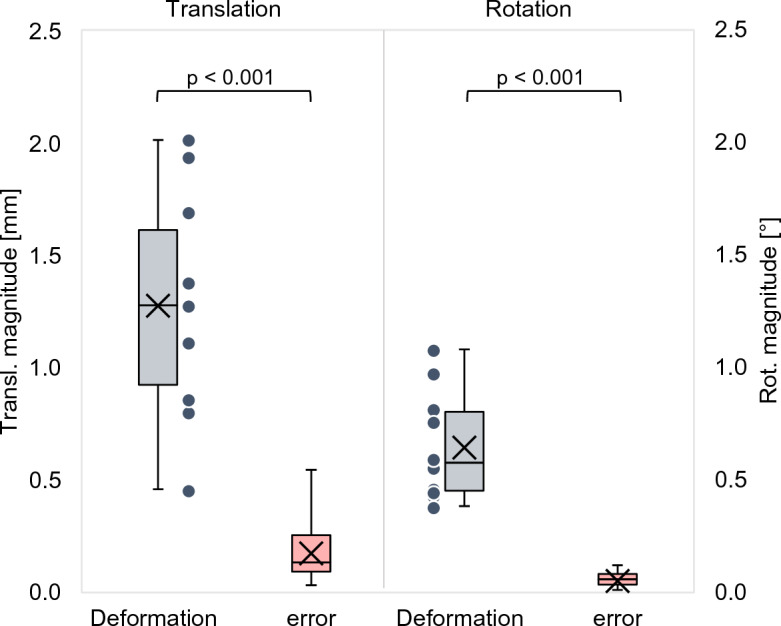
The grey box- and whisker plots show the relative translation- and rotation-magnitudes between the proximal and distal tibia in the cadaver group (cemented implants, 10 scans). The dots represent the individual results. The red boxplots show the methodological error. The p-values show the level of significance between the deformation and the methodological error.

Under the influence of mechanical loading, the tibia appeared to deform by 1.27 (± 0.50 mm) and 0.64° (± 0.25°, Fig. 6). This is true deformation since these displacements were statistically significantly (p < 0.001) larger than the methodological error in this experiment: 0.17 mm (± 0.12 mm) and 0.05° (± 0.03°).

**Figure 7:**
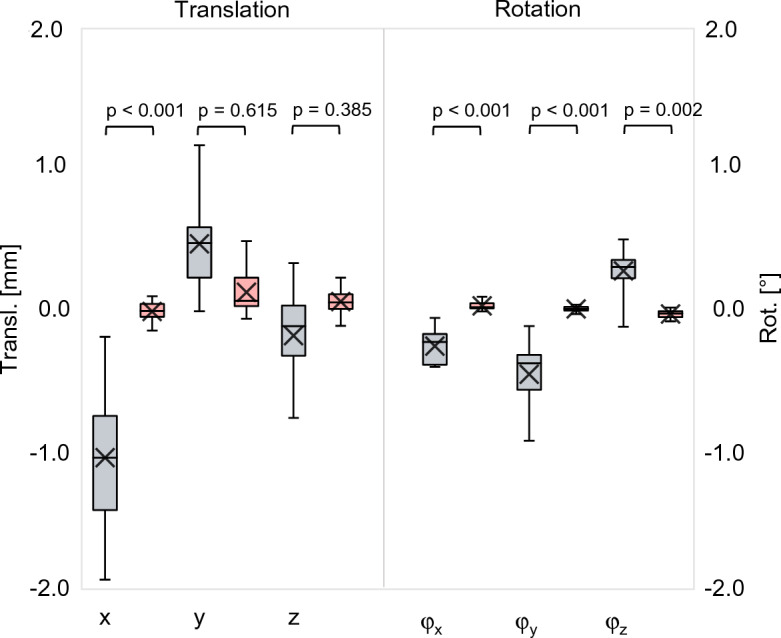
Boxplots (gray) showing tibia deformation decomposed in three translations along the x, y, z- axis and three rotations about these axes (φ_X_, φ_Y_, φ_Z_). The red boxes show the corresponding methodological error. The p-values show the level of significance between the deformation and the methodological error.

By decomposing the deformation displacement into translations along the x-, y- and z-axes of the local coordinate system (Fig. 4) and rotations about these axes (Fig. 7), the largest translation is observed in the x-direction (− 1.06 mm (± 0.50 mm)) and the largest rotation about the y-axis (− 0.47˚ (± 0.26°)). This was to be expected given the direction in which the mechanical load was applied (see Fig. 3). These are true translations and rotations since they are statistically significantly larger than the methodological error in this experiment (− 0.15 mm; p < 0.001 and − 0.04°; p < 0.001).

### Implant displacement under load

#### Measuring the level of implant displacement due to deformation

**Figure 8:**
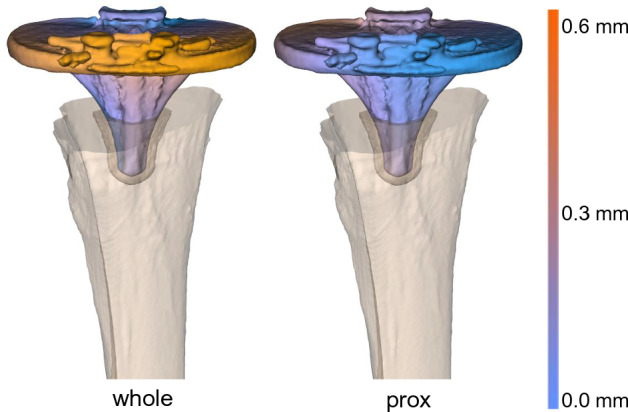
Heatmap showing that apparent implant displacement is increased by tibia deformation when measured with respect to the whole tibia (left) compared to the proximal tibia (right). One cadaver scan was selected for this example.

To visualize the effect of tibia deformation on implant displacement measurement, a displacement heatmap of one cadaver evaluation with a cemented implant is illustrated (Fig. 8). In this example, the maximum displacement between the implant and the whole tibia was approximately 0.60 mm, while between the implant and the proximal tibia, the maximum displacement was approximately 0.35 mm.

**Figure 9:**
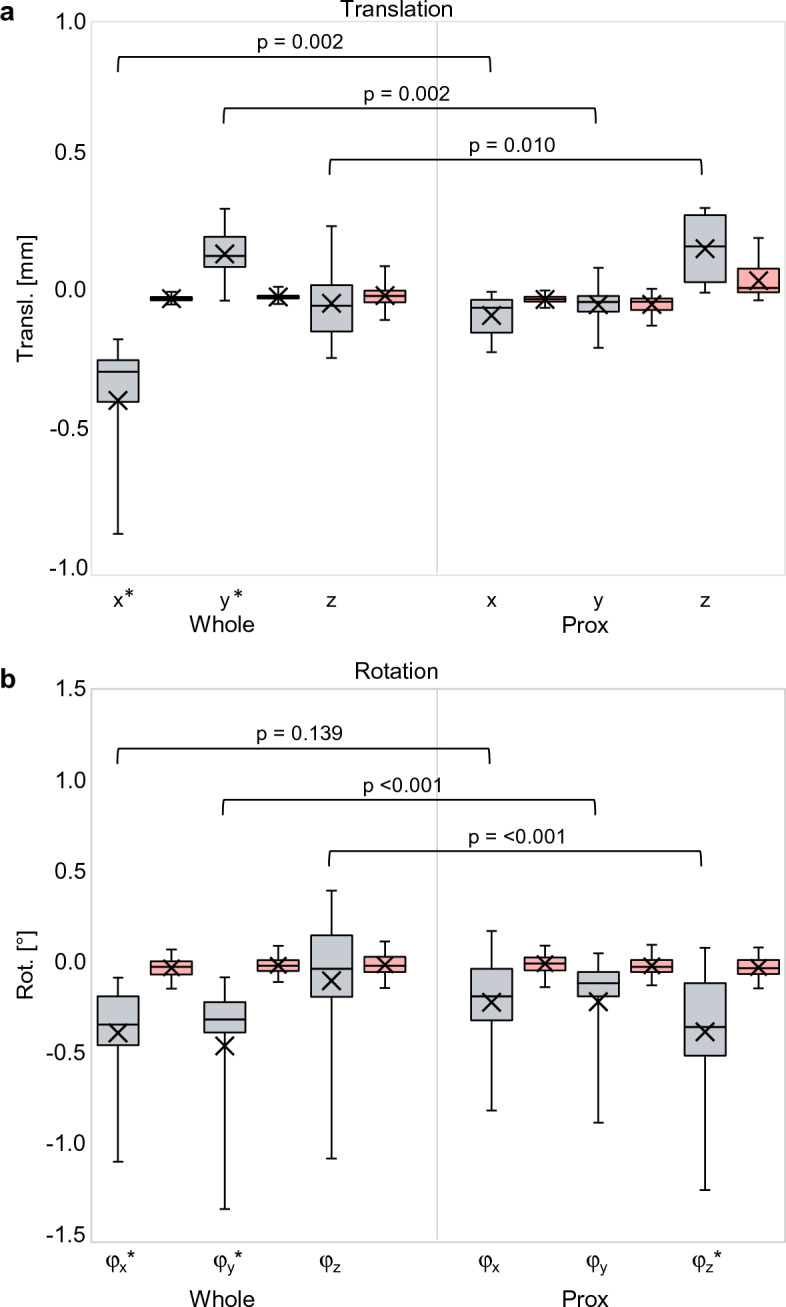
Boxplots showing implant translation (**a**) along the x-, y- and z-axis and implant rotation (**b**) about these axis (φx, φy, φz), measured between the fixed implant and the whole tibia (whole) and between the fixed implant and the proximal (prox) tibia. The red boxes show the methodological error in the corresponding direction. The p-values show the level of significance between the Whole and Prox tibia in each direction. (*) Statistically significant difference with the methodological error.

**Figure 10:**
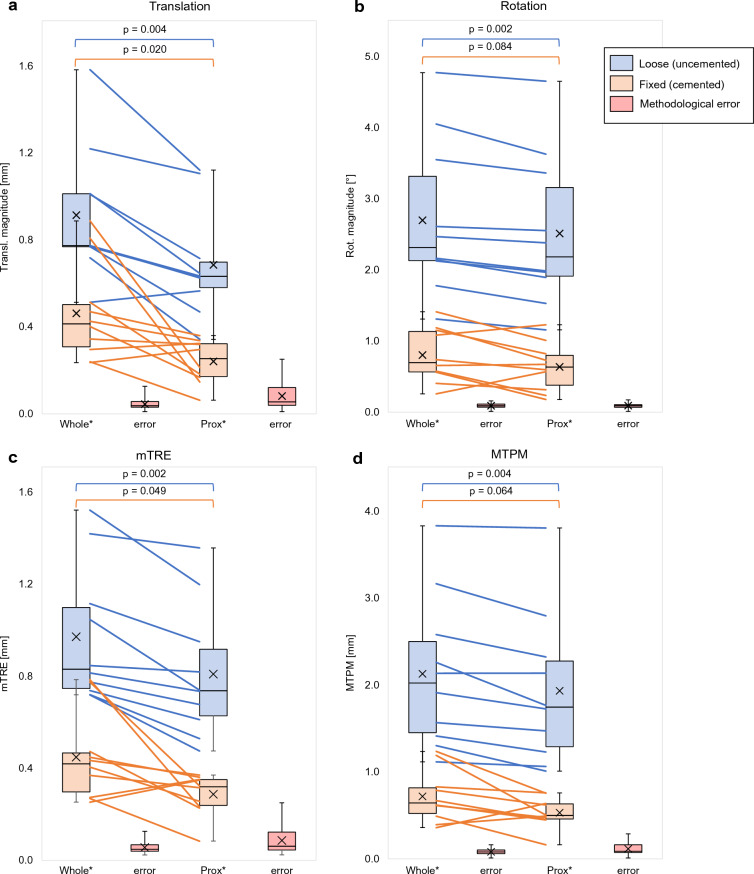
Boxplots showing the translation magnitude (**a**), rotation magnitude (**b**), mean Target Registration Error (mTRE) (**c**) and Maximum Total Point Motion (MTPM) (**d**) for the uncemented (in blue) and cemented (in orange) implants, measured with respect to the whole tibia (Whole) and the proximal tibia (Prox). The lines connect results obtained for the same leg, evaluated using the whole and proximal tibia. The p-values show the level of significance between using the Whole and Prox tibia, for both the uncemented and cemented implants. Each implant displacement parameter was statistically significantly larger than the methodological error, plotted in red (p < 0.001 for all measurements). (*) Statistically significant difference with the methodological error.

The effect of tibia deformation on measuring implant displacement in all ten cadavers with a cemented implant is demonstrated by the decomposed x, y, z translations and rotations of the fixed implant with respect to the whole and proximal tibia (Fig. 9). The other displacement parameters are shown in Fig. 10. The translation between the implant and whole tibia was statistically significant in the – x (p < 0.001) and + y direction (p = 0.007), which corresponds to the direction in which the tibia deforms (see Fig. 7). When using only the proximal tibia, the implant translation is no longer statistically significantly different from the methodological error (x: p = 0.615, y: p = 0.976), correctly indicating that the implant was fixed. This same trend was observed for the rotations about the x- and y-axes.

### Differentiation between loose and fixed implants

The implant displacement of loose (uncemented) and fixed (cemented) implants, calculated as the translation magnitude, rotation magnitude, mTRE and MTPM, was larger with respect to the whole than to the proximal tibia. By using the proximal tibia, no overlap between the uncemented and cemented implants was found for the mTRE and MTPM, indicating better differentiation between loose and fixed.

The translation magnitude (Fig. 10a) was statistically significantly smaller when using the proximal tibia compared to using the whole tibia, for both the cemented (whole: 0.45 ± 0.22 mm, prox: 0.23 ± 0.10 mm, p = 0.020) and uncemented (whole 0.91 ± 0.31 mm, prox: 0.68 ± 0.25 mm, p = 0.004) tibia. By using the proximal tibia, one case overlapped between uncemented and cemented, an improvement compared to using the whole tibia (3 cases of overlap). The rotation magnitude (Fig. 10b) was only statistically significantly different between using the whole and proximal tibia for the uncemented implants (whole: 2.69 ± 1.09˚, prox: 2.50 ± 1.07˚, p = 0.002), and not for the cemented implants (whole: 0.79 ± 0.38˚, prox: 0.63 ± 0.34, p = 0.084). One case of overlap between the loose and fixed cases was found for rotation in both approaches.

When quantifying implant displacement as the mTRE (Fig. 10c), a statistically significant difference between using the whole and proximal tibia was found for both the cemented (whole: 0.44 ± 0.18 mm, prox: 0.28 ± 0.09 mm, p = 0.049) and uncemented (whole: 0.96 ± 0.28 mm, prox: 0.80 ± 0.27 mm, p = 0.002) implants. When using the proximal tibia, no more overlap between the loose and fixed cases was found. The MTPM (Fig. 10d) was statistically significantly larger when using the whole tibia compared to using the proximal tibia in the uncemented implants (whole: 2.12 ± 0.82 mm, prox: 1.92 ± 0.11 mm, 0.004), and not in the cemented implants (whole: 0.71 ± 0.29 mm, prox: 0.52 ± 0.17 mm, p = 0.064). Again, using the proximal tibia improved differentiation between loose and fixed implants.

Compared to the methodological error plotted in red (Fig. 10), all implant displacement parameters were statistically significantly larger (p < 0.001) for both the whole- and proximal tibia, in the cemented and uncemented implants.

## Discussion

This paper shows that, as a consequence of tibial deformation under external valgus- and varus loading, tibial TKA implant displacement is overestimated if the entire segmented tibia is used as a reference. The deformation effect can be reduced by calculating implant displacement with respect to the proximal tibia instead of the whole tibia.

An advantage of using the proximal part is that it enables CT scanning of only the knee region instead of the entire tibia, resulting in a dose reduction. It further speeds up image analysis since less image data needs to be processed.

A disadvantage of using the proximal tibia as reference when calculating implant displacement is shown by the increase in methodological error, explained by the fact that image noise has a larger effect on registration when the segment is smaller. In addition, the proximal tibia at the level of the implant is more susceptible to metal artefacts which occur because of photon starvation and beam hardening^31^. These artefacts hamper precise segmentation in one scan and registration to the other. Improvements could be made regarding image acquisition and analysis. Optimization of the CT scanning protocol, for example by applying metal artefact reduction algorithms, may improve the image quality^32^. The image analysis may be optimized by exclusion of the tibia close to the bulky implant plateau, where metal artefacts are particularly pronounced. Additionally, an optimum may exist in choosing the length of the tibia segment, such that it is sufficiently large to reduce the methodological error while being short enough to keep the deformation effect acceptable. Whether these optimizations result in a significant improvement in the accuracy of the measurement of the looseness of the implant requires further research.

The centroid of the implant-plateau was used as the center of rotation, and is placed at the origin of the fixed local CS in the valgus image (Fig. 4). This origin was used to quantify implant translation. The translation depends on the location of the origin. For example, if the origin is placed at the point of the implant which shows the largest displacement, the translation magnitude would equal the MTPM. Selecting any other point on the implant results in a smaller translation. In our study, all implants were of the same brand and type. The choice of the origin therefore did not affect the variability of the measurement. In clinical practice, different implants (shapes and sizes) are used, which hinders inter-patient comparison of the implant translation. Implant rotation is independent of the location of the origin of the coordinate system on the object but is insufficient to describe the total displacement.

To enable inter-study comparisons for future determination of a loosening threshold, it would be convenient to have a scalar quantity that summarizes the complete implant motion. The MTPM can be best compared to measures of MTPM as measured by using model-based RSA, the current golden standard. A disadvantage of the MTPM is that it depends on a single point of the implant, which may be a point on a geometric artefact included in the segmentation. Also, MTPM scales with the object size, resulting in higher values for larger implants with the same rotation. The mTRE is less sensitive to artefacts since small and large values are averaged, but is also dependent on the implant size and proportions. Which parameter(s) allows robust inter-patient comparisons of implant displacement requires further research, in which clinical relevance, discriminative value, user-dependency (segmentation) and technical accuracy should be considered.

The accuracy and precision that was presented in the present work using the whole tibia as reference, seemed slightly better compared to the previously reported methodological error by our group^10^. This may be explained by the fact that in the current study segmentation of the tibia cortex was used, including the region around the implant stem, which resulted in more points for registration to the varus image than using points of only the outer contour below the stem, as in the previous study. Caution should be given in comparison of our displacement measurements and methodological error to reported literature since different definitions for accuracy and precision are used. For RSA-based studies an ISO 16087:2013 is established which defines precision by the proximity between repeated measurements under similar conditions^33^. This approach was used in different studies reporting CT-based motion analysis (CTMA) for evaluation of implant migration. The precision of MTPM for CTMA in tibial implant migration was recently evaluated in a porcine knee^34^. They calculated the mean and 95% confidence interval of 21 double examinations without migration in two CT scanners to be 0.04 mm (CI 0.03–0.12) and 0.11 mm (CI 0.04–0.19), which lies in range with our methodological error of both the whole- and proximal approach (whole:0.07 ± 0.03 mm, prox: 0.11 ± 0.06 mm). Two studies that evaluated hip cup migration using CTMA in patients reported a precision between 0.07 and 0.16 mm in translation and 0.21–0.37° in rotation^35,36^. Other experimental phantom studies of the hip and shoulder found the precision to range between 0.01–0.27 mm and 0.06–0.54^37–39^. Tibia bending under axial loading has been widely studied in context of understanding bone-mechano adaptions under compression and torsional deformations^40–42^. These studies show that tibia bending depends on both loading regime (for example load magnitude, direction, frequency) and bone characteristics, including geometrical parameters such as cortical thickness, bone mineral density and curvature. It was also shown that bones tend to bend more in unusual loading patterns^43^, that may apply to the valgus and varus loading as applied in the current research. A transverse loading pattern was tested in cadaver tibiae in context of obtaining mechanical properties^44^. However, quantitative comparison between these previous studies and the presented results in this paper is not possible due to different loading regimes.

A limitation of the present study is the use of a small number of cadaver specimens. The specimens had a median age of 82, which is generally older than patients eligible for implant revision and may contribute to a possible overestimation of tibia deformation. Also, as previously discussed^10^, the uncemented implant most likely gives an exaggeration of mobility, compared to the clinical situation where more overlap between displacement measurements of loose and fixed implants is expected. Another limitation is that all cadavers had the same implant. Implant design (geometry, material) affects the mechanical response to load-inducement^45^, increasing inter-patient variability of displacement measurements. This emphasizes the need to correct for the tibia deformation effect to calculate true implant displacement.

In this study, an alternative analysis method is evaluated whereby the proximal tibia is used in the calculation of implant displacement to minimize the effect of tibia deformation. Despite this correction, the implant displacement of the cemented (fixed) implant is statistically significantly larger than the methodological error for (Fig. 10). Local deformity of the proximal tibia and suboptimal cementation account for this. For future utilization, it would be interesting to investigate a correction for local (epiphyseal) deformation to further improve the non-invasive measurement of implant loosening.

## Conclusion

Under the applied valgus-varus load for CT-based tibial implant loosening assessment of a total knee arthroplasty, the tibia deforms. This deformation causes an overestimation of the true displacement between the implant and bone. By using only the proximal part of the segmented tibia as the reference object, the effect of tibia deformation can be reduced, which improves the differentiation between uncemented (loose) and cemented (fixed) implants in cadavers.

### Supplementary Information

Supplementary Information.

## Data Availability

All data generated or analyzed during this study are included in this published article (and its Supplementary Information files).
